# Exome sequencing combined with semantic discovery identifies strong disease-associated candidates in a single case of relapsing remitting multiple sclerosis

**DOI:** 10.1186/1753-6561-6-S6-P10

**Published:** 2012-10-01

**Authors:** Mahjoubeh Jalali Sefid Dashti, Maritha Kotze, Susan Janse van Rensburg, Alan Christoffels, Junaid Gamieldien

**Affiliations:** 1 South African National Bioinformatics Institute, University of the Western Cape, Bellville, 7535, South Africa; 2 Department of Pathology, University of Stellenbosch, Tygerberg, 7505, South Africa

## Background

As known disease-associated variants identified through large cohort-based studies often explain only a small percentage of genetic risk in multifactorial disorders such as multiple sclerosis (MS), alternative methods for identification and prioritization of variants that directly and/or indirectly play a role in disease development have become increasingly important. We were tasked with identifying possible genetic causes in a case of atypical relapsing remitting MS (RRMS) that also presented with porphyria-like symptoms and where demyelination was halted in the patient upon iron supplementation. As the patient had no parents or siblings that could be used as references for filtering exome variants, we aimed to develop a new prioritization strategy based on the combination of a predicted deleterious effect on the protein and existing knowledge of the biological roles of the genes and their contribution to relevant phenotypes.

## Materials and methods

Exome sequencing was performed and functional SNP analysis and frameshift indel detection were carried out using a combination of prediction tools. Predicted deleterious variants were further assessed with respect to their possible involvement in MS using our internal biomedical semantic database, the B.O.R.G. (BioOntological Relationship Graph), which integrates existing biomedical knowledge and uses path-based graph theoretic querying to discover links between biological concepts. The semantic model used in this study incorporated known human, mouse and rat: gene, gene-to-disease, gene-to-phenotype, gene-to-function and gene-to-pathway and orthology relationships in conjunction with 'surrogate' phenotype and biological function links to the relevant disease term.

## Results

The exome sequence resulted in identification of 64,890 variants based on the human reference genome, of which 4,847 missense variants were predicted to have a damaging effect. These were used to interrogate the semantic network, which simplified exploring the network for transitive relationships that may explain the biological contribution of identified mutations to the development of disease, either directly from human evidence or transitively via model organism evidence such as knockout phenotypes. 750 variants were found to be potentially involved in MS based on known gene-to-disease associations or via surrogate-phenotype, -function or -pathway links to MS. Examples of strong candidate MS genes identified were: *BACE1*, *HEXB* and *NRCAM* (involved in myelination), *CNTN2* (involved in axon development), and *CCM2* (implicated in immune response and central nervous system inflammation). In addition, we identified deleterious variants in *IREBP* and *CYBRD1*, which are involved in iron regulation and homeostasis, and may contribute to the iron deficiency condition of the patient. Several other strong candidates are being evaluated further.

## Conclusions

While the list of candidates obtained from this study is quite large, a number of them may in fact represent a part of the large number of variants proposed to be associated with the 'missing' (approximately 80%) MS genetic risk, with each adding a tiny percentage to the overall risk of developing the disease. As these variants fulfil many criteria, our knowledge-driven prioritization strategy may appear to have the potential to improve discovery of causative variants in non-Mendelian diseases and also rare diseases where large cohorts are almost impossible to build.

